# Recruitment and retention of mothers of preschoolers and school-aged children in a social media-delivered healthy eating intervention: lessons learned from a randomized controlled trial

**DOI:** 10.1186/s13063-020-04628-0

**Published:** 2020-08-10

**Authors:** Audrée-Anne Dumas, Simone Lemieux, Annie Lapointe, Véronique Provencher, Julie Robitaille, Sophie Desroches

**Affiliations:** Institute of Nutrition and Functional Foods, School of Nutrition, Faculty of Agriculture and Food Sciences, Université Laval, Pavillon des services, office 2729-P, 2440 Hochelaga Boulevard, Quebec City, Quebec G1V 0A6 Canada

**Keywords:** Social media, Blogs, Healthy eating, Recruitment, Retention, Randomized controlled trial, Mothers

## Abstract

**Background:**

Social media represent cost-effective platforms to advance the dissemination and uptake of health research to improve population health. However, there is limited evidence available to support researchers overcome methodological challenges related to recruitment and retention of participants in studies using social media for delivering behavior change interventions. This study aims to describe the recruitment and the retention strategies used in a randomized controlled trial (RCT) that evaluated a blog-delivered healthy eating intervention among mothers of preschoolers and school-aged children.

**Methods:**

Eighty-four adult mothers of children aged between two and 12 years old living in Quebec City, Quebec, Canada, were recruited between October 2015 and February 2017 using traditional methods (e.g. institutional email lists, flyers, newspapers, and word of mouth) and Facebook advertisements. Retention rates were calculated at the end of the 6-month intervention and at a 12-month follow-up assessment. Sociodemographic characteristics, Internet use behaviors and retention rates of mothers recruited through traditional methods and Facebook were compared using Wilcoxon-Mann-Whitney tests and Fisher exact tests.

**Results:**

Of the 196 mothers who responded to the recruitment call, 87 (44.4%) were eligible and 84 (42.9%) were randomized to the trial, representing a recruitment success of 76.4% (84/110) from the planned sample size target. Among those, a minority (3.6%) were recruited using Facebook. Those mothers presented similar sociodemographic characteristics to those recruited using traditional methods. Retention rates were 73.8% and 66.7% at 6 and 12 months, respectively, with similar rates between mothers recruited using Facebook and traditional methods. Various challenges associated with population retention were highlighted with lack of time being mothers’ main reason for withdrawing from the study.

**Conclusions:**

The methodological challenges experienced during the conduct of the blog-delivered healthy eating intervention allowed to draw upon several lessons regarding the recruitment process and the retention of mothers of preschoolers and school-aged children to inform future social media-delivered research. Recommendations for future research include exploring mothers’ perceptions and preferences to tailor social media recruitment, ensure that interventions are delivered to them using social media platforms that are already integrated into their routine, and are providing remote outcome assessments to increase participant retention.

**Trial registration:**

Clinical Trial Protocol NCT03156803. Registered on 17 May 2017, retrospectively registered.

## Background

Social media usage has greatly increased in all segments of the population, including older adults and low-income communities [1]. In the USA, the use of social media has increased from 5 to 69% between 2005 and 2018 [2]. These platforms have triggered a revolution in the way individuals and patients access and use health information, as well as how they have become active players in maintaining their health and managing diseases [3]. Indeed, the use of social media by patients and health professionals has been widely described in the scientific literature [4–6]. Various social media platforms are available for healthcare professionals, such as collaborative projects (e.g. wikis), blogs and microblogs (e.g. Twitter), and social networking sites (e.g. Facebook), to improve patient care, patient education, and health promotion among the population by disseminating actionable messages [7, 8] and complementing face-to-face clinical counseling to support healthy behavior change efforts [9–11].

Leveraging this trend, researchers have been exploiting social media platforms to recruit participants [12–14], undertake online data collection [15], and deliver behavior change interventions [11, 16, 17]. In particular, some evidence suggests that Facebook advertisements may be an efficient and cost-effective method for recruiting mothers of young children into social media-delivered public health research programmes [18–20], by allowing the personalization of advertisement settings to specific demographics and interests of users. Using Facebook may be an ideal choice for recruiting millennial mothers (millennials are defined by the Pew Research Center as anyone born between 1981 and 1996, thus aged 24 to 39 in 2020 [21]) due to their high level of engagement with this social media platform in their everyday lives [22]. From a public health perspective, mothers of preschoolers and school-aged children represent an important target audience for social media-delivered healthy eating promotion interventions due to the high influence they exert on their children’s dietary behaviors and risk of obesity [23–27]. In many societies, it is still a lingering gender role expectation that the families’ dietary decisions fall into women responsibilities [28]. As a consequence, mothers have life priorities and face challenges, such as family engagements and time constraints that may jeopardize their abilities and willingness to participate in clinical trials, but such situation may be minimized in Internet-delivered interventions [29]. Some examples of social media-delivered interventions targeting mothers have been described in the literature [19, 30–33], but few detailed reports [20, 34] are available on the recruitment and the retention of mothers of preschoolers and school-aged children.

Randomized controlled trials (RCTs) have become the gold standard methodology to generate evidence-based conclusions about the effectiveness of most interventions [35]. To date, exploiting such high-quality design comes, however, with several difficulties associated with recruitment, compliance, and retention of study participants. For instance, reaching original recruitment targets is a common challenge for researchers conducting RCTs, which may have deleterious consequences such as underpowered studies, expensive deployment of resources, and, in some cases, limiting the research capacity to evaluate the impacts of health interventions [36, 37]. Thus, a priority for research pertaining to trial methods is to find efficient and effective ways to improve recruitment rates.

The objectives of this paper were therefore (1) to compare the recruitment rates of traditional methods and Facebook advertisements used to recruit mothers of preschoolers and school-aged children in a blog-delivered healthy eating intervention, (2) to describe the characteristics of mothers recruited through traditional methods and Facebook, and (3) to describe the retention rates according to specific recruitment methods in order to provide recommendations to inform future research.

## Methods

### Study design and participants

This study is based on secondary, post-hoc analyses from a parallel, randomized, controlled trial conducted from January 21, 2016, to September 7, 2017, which evaluated the effects of an evidence-informed healthy eating blog on eating habits and behaviors of mothers of preschoolers and school-aged children. The full trial protocol [38] and the effects of the intervention [39, 40] have been previously described. The original study protocol was approved by the Université Laval Research Ethics Committee (project no 2014-257 A-5/12-07-2016). In the present analyses, as in the main trial, eligible participants were women aged 18 years or over recruited in Quebec City, Canada, between November 11, 2015, and February 10, 2017. Mothers were eligible if they had at least one child aged between two and 12 years, were primarily responsible for food purchase and preparation in the household, consumed fewer than the recommended daily servings of vegetables and fruit and/or of milk and alternatives food groups as described in the 2007 edition of Canada’s Food Guide [41] (i.e. fewer than seven servings per day of vegetables and fruit and/or less than two servings per day of milk and alternatives), and had an Internet access. Mothers taking medications that could affect food intake, having an eating disorder, currently dieting, pregnant, or breastfeeding at the moment of recruitment were excluded.

### Recruitment procedures

In the context of the main trial, a three-phase intervention was conducted, with identical intervention content and sequence for each phase, to reduce the delay between recruitment and the start of the trial. Recruitment periods for the three experimental phases were conducted subsequently in different periods of the year (i.e. winter through summer; fall through spring; and spring through fall). For the first experimental phase, the recruitment period occurred between October and December 2015. Traditional electronic media were used (i.e. advertisements to an email list of people that had indicated their interest to participate in the research institute’s clinical studies and to email lists of Université Laval employees and students) as well as word of mouth. The recruitment period for the second experimental phase occurred between July and September 2016. In addition to traditional electronic media used for experimental phase one, we used concurrently an advertisement displayed for 3 weeks in the Intranet of the 13,500 employees working in a five-hospital network in Quebec City, Canada; flyers posted in community centres targeting families, schools, and day-care centres; personalized emails sent to mothers ineligible to participate in a web-based family nutrition intervention study [42]; and advertisements on Facebook. A Facebook ad was created using a combination of wording and images to target French-speaking females aged 18 years or over, living within 40 km from Quebec City centre, Canada, who had at least one child aged 12 years or younger, and whose personal profile included interests related to food and nutrition. The Facebook ad was online for a period of 7 days from September 13, 2016, to September 20, 2016. Interested mothers clicked on the ad and were automatically directed to the research institute website containing a brief description of the study and eligibility criteria as well as contact information to reach the research coordinator of the study. Additionally, a total of 10 status publications were published at non-periodic times on a public Facebook page of the study created on June 2, 2016, and the study Facebook page was advertised on private Facebook groups of the dietetic association and two summer camps for school-aged children in the province of Quebec, Canada. Last, recruitment for the third experimental phase occurred in January and February 2017 using the traditional electronic media used for experimental phase one, two additional status publications on the public Facebook page of the study, as well as one advertisement in a local newspaper that was displayed electronically on the newspaper website and in print.

Interested mothers contacted the research coordinator or a graduate student who explained the study objectives and confirmed participants’ eligibility through a phone interview. During the screening process, mothers were asked how they had heard of the study. Interested mothers meeting all eligibility criteria were invited to attend a clinical appointment at the research institute to read and sign an informed consent form and perform baseline outcome assessments prior to randomization in the intervention.

### Sample size

In the context of the main trial, a sample size of 82 mothers was estimated based on findings from a previous intervention study [43] to allow the detection of a 28% difference in vegetable intakes, 6 months after the end of the intervention (*T* = 12 months), with a standard deviation of 2.05 in servings of vegetables, a power of 0.95, and a two-sided 0.05 significance level. Because attrition rates have been shown to vary between 6% and 75% in social media-based interventions in dietetic practice [16], we anticipated an attrition rate of 25% and therefore planned an original recruitment target of 110 mothers. However, as previously reported [39], to avoid losing participants who were waiting for the intervention to begin, recruitment procedures were prematurely ended when 84 eligible mothers had been recruited due to difficulties in recruiting mothers.

### The healthy eating blog trial

In the context of the main trial, two experimental conditions were compared: a 6-month dietary intervention exclusively delivered through an evidence-informed healthy eating blog with a weekly posting written by a registered dietitian (RD) to promote increases in vegetables and fruit, and milk and alternatives consumption (BLOG group), and a control condition with no access to the intervention blog. Reporting of this trial followed the Consolidated Standards of Reporting Trials 2010 (CONSORT) (Additional file 1). The extensive description of the intervention blog development process was previously reported [38]. In summary, an evidence-informed blog designed in accordance to the preferences of female social media users [44, 45] was written and monitored during a 6-month intervention period by a 5-year experienced RD blogger. Blog posts were published once a week and targeted one objective per month inspired by Health Canada Eat Well Campaign [46], as well as perceived barriers of mothers to healthy eating [47–49]. Theory-based intervention methods [50, 51] (e.g. modeling, goal setting, and provision of feedback on performance) were translated into practical applications on the blog. Mothers were encouraged to visit the blog at least once a week and to submit questions and testimonies to the comments section of the blog. Engagement promoting methods [45, 52] were used to improve participant retention (e.g. peer and counsellor support, emails announcing weekly updates to the blog, as well as the use of a narrative approach for writing blog posts and prompt responses to participants’ comments). The control group was a waiting-list control condition. During the 6-month intervention period, the control group did not access the blog nor were contacted by the RD-blogger, but met the research coordinator at the research institute for all outcomes’ assessment appointments. After the last outcome assessment, the control group was granted access to the blog’s archives.

During the main trial, mothers performed dietary outcome assessments remotely using web-based questionnaires after 3 months, at the end of the 6-month intervention, and after a 6-month follow-up (*T* = 12 months). In-person appointment times were scheduled at convenient times for mothers for body weight measurement at six and 12 months. Mothers self-reported their body weight at 3 months to reduce the burden associated with outcome assessment. For all in-person outcome assessment appointments, parking fees were reimbursed, and mothers had the option of being accompanied by their children to avoid the need to make arrangements for childcare.

### Outcome assessment

#### Sociodemographic variables and blog use habits

Sociodemographic characteristics (i.e. age, ethnicity, highest education level completed, working status and marital status, annual family income, number and age of children) as well as their Internet and blog use habits were collected using a self-administered Web-based questionnaire completed by mothers at baseline.

#### Anthropometric measures

Body weight was measured to the nearest 0.1 kg in light clothing without shoes (BWB-800S Digital scale, Tanita) during in-person clinical appointments at the research institute. Height (baseline only) was measured to the nearest millimetre with a stadiometer (Seca 222 Mechanical Telescopic Stadiometer) without shoes. Waist circumference measure was also taken to the nearest millimetre following standardized procedures [53].

#### Facebook advertisement metrics

The performance and reach metrics of the Facebook advertisement were assessed using Facebook Audience Insights [54].

### Statistical analysis

The number of enquiries from eligible mothers who were randomized to the trial and from those who completed the study for each recruitment strategy was described using percentages. To determine whether and how mothers recruited via Facebook compared to those recruited through traditional methods, the baseline sociodemographic and anthropometric characteristics and Internet use behaviours of each group were compared using Wilcoxon-Mann-Whitney tests and Fisher exact tests due to the non-normal distribution of the data and unequal sample sizes of the two groups. Last, the characteristics of mothers who completed the study and those who withdrew from the study at 6 months and at 12 months were compared using chi-square and independent-sample Student *t* tests. Analyses were performed using SAS University Edition (SAS Institute Inc., Cary, NC, USA). The critical alpha value for statistical significance was established at .05.

## Results

### Flow of participants from recruitment to randomization

**Fig. 1:**
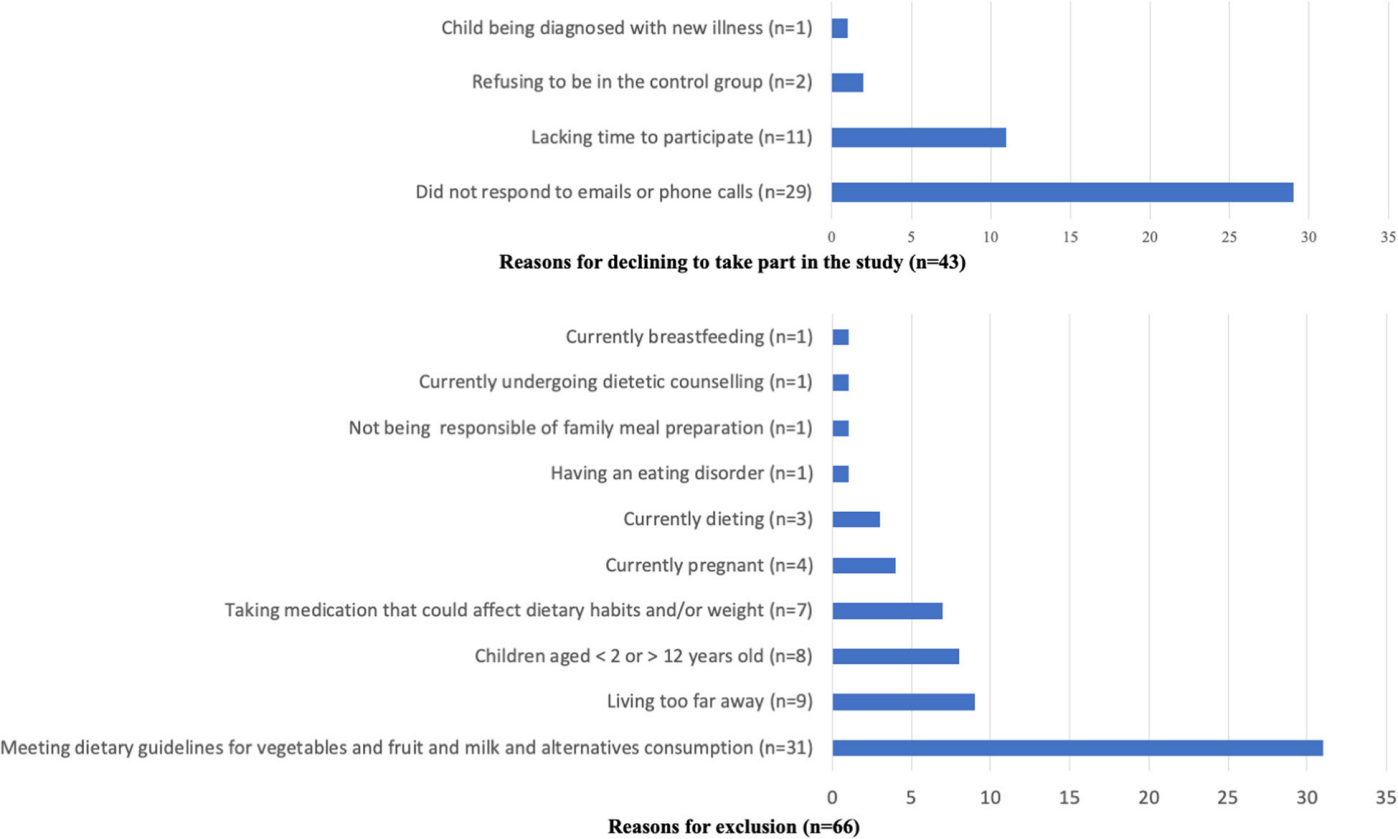
Mothers’ reasons for declining to take part in the study and for exclusions

Table 1 compares the proportions of mothers who were screened for eligibility and who were randomized to the study according to recruitment strategies (i.e. social media [Facebook], traditional electronic media [institutional email lists, hospital employee Intranet], print media [flyers, newspaper advertisements], other [e.g., word of mouth, summer camps]). A total of 196 mothers responded to the recruitment call. Recruitment using traditional electronic media generated more than half of the total number of enquiries, followed by Facebook advertisements. Twenty-nine mothers never replied to emails or phone calls after they had first contacted the research coordinator for participating in the trial, among whom one third were mothers reached by Facebook advertisements (9/27, 33.3%) and only 16.9% (20/119) of mothers had been reached by traditional electronic media. Among the interested mothers, 43 declined to participate in the study after they received full information about the nature of their participation and 66 did not meet the eligibility criteria. Among the 87 eligible mothers, three did not attend their first in-person outcome assessment appointment, resulting in a final sample of 84 mothers randomized to the study for a recruitment success of 76.4% (84/110) from the planned sample size. The majority of the final sample had heard of the study through an institutional employees and students email list. Reasons for not taking part in the study and the reasons for exclusion are presented in Fig. 1.

**Table 1:** Total enquiries, eligible, and randomized participants per recruitment strategy

Modality	Recruitment strategy	***N*** enquiries	(%)	***N*** eligible	(%)^**1**^	***N*** randomized	(%)^**1**^
**Social media**	**Facebook**	**27**	**(14)**	**4**	**(15)**	**3**	**(11)**
**Traditional electronic media**	University email list	66	(34)	43	(65)	41	(62)
	Research institute email list	29	(15)	14	(48)	14	(48)
	Hospital employee Intranet	24	(12)	11	(46)	11	(46)
	**Subtotal**	**119**	**(61)**	**68**	**(57)**	**66**	**(55)**
**Print media**	Flyers	1	(1)	0	(0)	0	(0)
	Local newspaper advertisement	6	(3)	6	(100)	6	(100)
	**Subtotal**	**7**	**(4)**	**6**	**(86)**	**6**	**(86)**
**Other**	Word of mouth	4	(2)	1	(25)	1	(25)
	Summer Camp	1	(1)	1	(100)	1	(100)
	Recall list of other family-based nutrition intervention study	17	(9)	4	(24)	4	(24)
	**Subtotal**	**22**	**(11)**	**5**	**(23)**	**6**	**(27)**
**Not described**	**Not described**	**21**	**(11)**	**3**	**(14)**	**3**	**(14)**
**Total**		**196**		**87**		**84**	

^1^Percentages are expressed as the proportions of eligible or randomized mothers from the number of enquiries for each recruitment method.

### Performance, cost, and reach of the Facebook recruitment

**Fig. 2:**
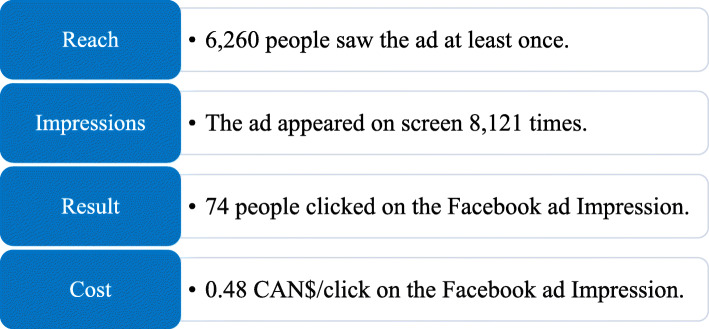
Facebook advertisement data over a period of 7 days. Values were estimated by Facebook Audience Insights [54]. Impressions measure how often the ad appeared on the screen of the target audience

Figure 2 presents the performance data and cost related to the Facebook advertisement. A 7-day display of the ad cost 34.57 CAN$. Almost half (36/74, 48.6%) of the estimated number of people who clicked on the Facebook ad carried through to the screening process for the study. However, Facebook was not considered a cost-efficient recruitment strategy as only three mothers recruited using Facebook were randomized in this study.

Twelve status publications were posted at non-periodic times on the public Facebook page of the study, which had a potential reach of 5089 Facebook users (Table 2). Among those, 9.2% (469/5089) engaged with the Facebook posts by either sharing them to other users, liking, or commenting the publication content (e.g. suggesting the name of a friend who could be interested in taking part in the study) (Table 2).

**Table 2:** Reach and user engagement during the Facebook recruitment^a, b^

Performance criteria	***n***
Total reach of Facebook posts (unique users)	5089
Total impressions of Facebook posts	8974
People engaged (e.g. share, likes, comments, clicks) with the Facebook posts (unique users)	469
Total clicks on the Facebook page	639
Unique users who liked and clicked on the Facebook page	92

^a^Valued estimated by Facebook Audience Insights [54].

^b^Impressions measure how often the ad appeared on the screen of the target audience.

### Comparison of mothers recruited using Facebook versus traditional strategies

Facebook advertisements attracted mothers (*n* = 3) who had younger children (Facebook, mean ± SD = 3.3 ± 1.5 years old; traditional methods, mean ± SD = 8.0 ± 3.2 years old; *P* = 0.03) compared to those recruited using traditional methods (*n* = 78). There was no statistically significant difference between the groups regarding baseline sociodemographic characteristics, body weight, waist circumferences as well as their Internet use behaviours (Additional file 2).

### Retention of study participants

**Fig. 3:**
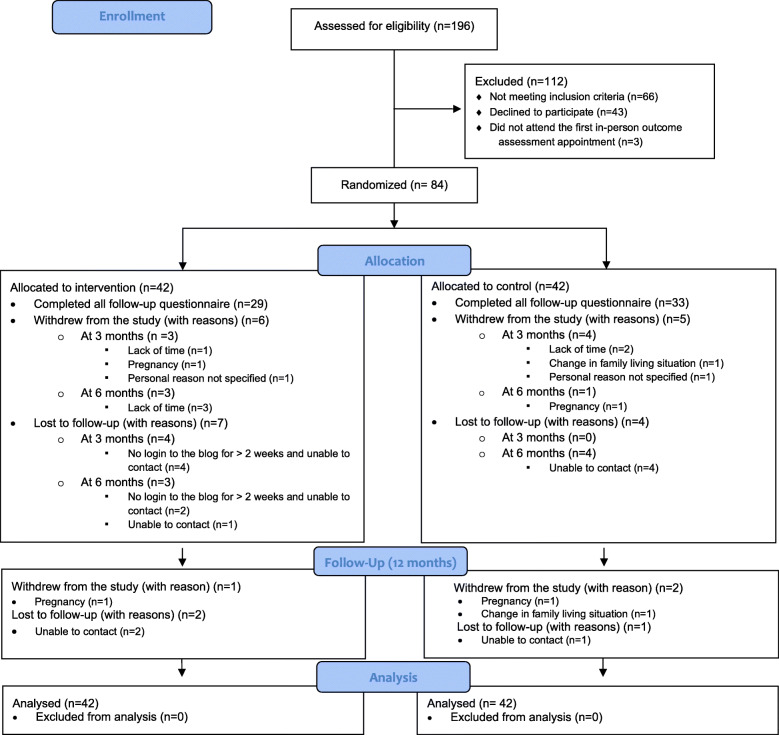
Consolidated Standards of Reporting Trial 2010 (CONSORT) flow diagram of the study

Figure 3 presents the flow of participants in the study. Of the 84 mothers randomized to the study, 62 completed the 6-month dietary intervention (73.8%) (BLOG group: *n* = 29; control group: *n* = 33) and 56 completed the follow-up outcome assessment at 12 months (66.7%) (BLOG group: *n* = 26; control group: *n* = 30). The main reasons for withdrawal were lack of time, new pregnancy, and unforeseen changes in family living situations. In total, the research team was unable to contact 11 participants for outcome assessment at 6 months and 3 participants for outcome assessment at 12 months. Among those, 6 mothers randomized to the BLOG group were lost to follow-up prior to the end of the 6-month intervention because they did not log on to the blog for more than 2 weeks despite email and phone reminders.

At 6 months, one third of mothers recruited using Facebook (1/3, 33.3%) and 25.64% (20/78) of those recruited using traditional methods discontinued their participation in the study prior to the end of the 6-month intervention. One third of mothers recruited using traditional methods (26/78, 33.3%) did not complete the 12-month follow-up assessment. The remaining mothers recruited using Facebook (2/3; 66.7%) completed both post-intervention and follow-up outcome assessments.

Mothers who discontinued participation prior to the end of the 6-month intervention (*n* = 22, 26.8%) had fewer children in their care (*P* = 0.02) and earned lower family income (*P* = 0.02), and a smaller proportion of them had read a blog prior to the start of the study (*P* = 0.01) compared to those who completed the intervention. Mothers who worked or studied full-time showed similar retention rates at 6 months to those who worked or studied part-time or who were unemployed (*P* = 0.34). Similar differences were observed between mothers who withdrew from the study prior to the 12-month follow-up outcome assessment (*n* = 28, 33.3%) and those who completed the 12-month outcome assessment (Additional file 3).

## Discussion

### Principal findings

This article detailed the recruitment and retention of mothers of preschoolers and school-aged children in a blog-delivered healthy eating intervention. Despite intensive efforts, the planned sample size was not reach and the recruitment prematurely ended to limit the risk of losing participants who were waiting for the intervention to begin. The methodological challenges experienced during the conduct of the trial allowed to draw upon several lessons regarding the recruitment process and the retention of participants to inform future social media-delivered research.

### Lessons learned from the recruitment process

#### Lesson #1: Social media-delivered dietary interventions should use recruitment strategies that are optimized for reaching socioeconomically disadvantage women

Recruiting fewer participants from the original target is a major challenge facing researchers conducting RCTs [36, 37], and this substantially attenuated the statistical power to detect differences in the primary outcomes between the two experimental groups in this trial [39]. Greater than anticipated human and financial resources had to be deployed to recruit eligible mothers. The blog intervention generated great interest among mothers with optimal dietary eating habits—as shown by the main reason for mothers’ ineligibility which was meeting the recommended daily servings of vegetables and fruit and milk and alternatives food groups—who were educated above high school level (college or university) and earned a high annual family income. These findings suggest that the recruitment strategies used in this trial were not optimized for reaching socioeconomically disadvantage women as well as those having suboptimal dietary habits. This may be due, in part, by a digital divide attributed to socioeconomic status, as research has shown that lack of Internet access is a barrier to taking part in Web-based studies [14], notably for adults with a low socioeconomic status living in Quebec City, Canada [55]. In the province of Quebec, it was estimated that 59% of low-income households had Internet access at home, which was statistically less than the average Internet penetration (90%) in 2017 [56]. Although the use of social media in the context of personal Internet use is growing in all segments of the population, including adults earning less than 20,000 $CAN per year in Quebec [57], there is a need to better understand the extent to which social media-delivered dietary interventions generate interest among socioeconomically disadvantage populations who are at higher risk of suffering from to this digital divide to reduce health inequities. Some studies have demonstrated success for the recruitment of socioeconomically disadvantage populations, such as overweight or obese low-income pregnant women [58] and obese, ethnic minority, low-income postpartum women [33], in social media-delivered health promotion interventions (e.g., for weight loss). In particular, advertisement through general practitioners working in local community centres that provide nutritional aid programmes among less privileged population groups during the recruitment process has been successful to recruit vulnerable women [58, 59]. Future qualitative research should be dedicated to understanding the barriers and facilitators influencing low-income women to participate in a social media-delivered dietary intervention and how recruitment strategies could optimize the reach and retention rates of low-income populations in such interventions.

#### Lesson #2: Social media-delivered dietary interventions targeting mothers should be designed with the goal of minimizing the burden of time associated with participation

The most frequent reasons for refusing to take part in the study related to practical inconveniences, such as time investments and distance to the study site, and related to the sustained participation during the 6-month intervention and commute for in-person outcome assessments that were required in this trial. Those barriers have also been identified as reasons why pregnant women would not participate in clinical research [60]. Those barriers to participation are not surprising given that mothers of young children have busy work-family schedules and high childcare responsibilities, which do not appear to be attenuated by the digitally remote mode of delivery of the intervention blog. This is further supported by the dissimilarities in recruitment between the results of this study and those of a previous study [11]—where being a mother of children aged between two and 12 years old was not an inclusion criteria—in which no recruitment difficulties were experienced in a blog-delivered healthy eating intervention for a period of 6 months among adult women. To further adapt these interventions to mothers of preschoolers and school-aged children, future studies should seek to identify their perceptions and preferences regarding what would be an acceptable design for a healthy eating intervention delivered through a blog or other types of social media platforms, such as the minimum engagement with the intervention blog that would be required for behaviour change support [61]. In addition, the timing and ways to conduct outcome assessments remotely (e.g. providing participants with a Bluetooth-enabled weight scale [62]) should also be explored as a strategy to enhance retention in future research.

#### Lesson #3: Social media recruitment must be planned attentively and tailored for the target population

Nine different recruitment strategies—ranging from traditional recruitment methods such as institutional email lists and newspapers, to more interactive strategies such as Facebook advertisements—were chosen to increase the visibility of the trial in places where potentially interested mothers would be looking. Unfortunately, limited efforts were invested in Facebook recruitment, and that resulted in a small number of eligible mothers who were enrolled using this method. Studies have demonstrated the efficacy of social media (e.g., Facebook, Twitter, YouTube) for increasing research dissemination and recruitment of various types of participants, such as doctors and medical students [63], young adults [64], older adults [15], and women trying to conceive [65], with some evidence of similar sociodemographic characteristics between samples recruited from in-person methods and those recruited though social media [65]. In these examples of studies, social media recruitment strategies had been attentively planned and re-evaluated, if necessary, during recruitment. Other intervention studies in which social media recruitment was shown effective offered a monetary incentive to increase participation responses [66], which was not the case in the present trial. Scholars have proposed best practices for using social media to recruit participants in medical research [67]; yet, even thoughtfully planned social media-enabled strategies are not necessarily more effective than other recruitment methods [68]. A review of medical studies using social media recruitment has shown that only 12 out of 30 studies found social media (mostly Facebook) to be more effective than traditional recruitment methods, among which only 4 were intervention studies [13]. Facebook attracted several mothers who were living too far away from the research institute to allow convenient travels for study appointments. Conducting outcome assessments remotely might have improved the Facebook recruitment rate and balanced the geographic representation of recruited mothers, and there is evidence that social media recruitment is most efficacious when used alongside online data collection [15]. Additionally, it remains unclear how many repeated viewings of social media advertisements are necessary before users take the next step towards engagement [67]. It is possible that Facebook recruitment rates could have been higher if the intervention had been delivered using this platform instead of a blog. It must, however, be acknowledged that similar participant retention rates were observed between the traditional recruitment strategies and Facebook. Nonetheless, capturing social media preferences and concerns of the target population should be a critical primary step to address in future research to tailor appropriate social media recruitment to capture the attention and interest of eligible individuals (e.g. choosing relevant keywords that are reflective of the interests of the target users’ profiles, regularly monitoring the recruitment campaign through Facebook analytics to flag advertisement parameters needing adjustments) and to avoid trial-and-error processes to determine the best recruitment yield [15, 67].

### Lessons learned for the retention of study participants

#### Lesson #4: Social media-delivered dietary interventions should be disseminated through platforms integrated into the target population’s social media routines

At completion of the intervention and at the 12-month outcome assessment, attrition rates were higher compared to previous social media-delivered lifestyle interventions targeting mothers [30, 31, 33, 69]. However, to the best of our knowledge, this trial is the first to have investigated the impact of a blog-delivered healthy eating intervention on dietary habits in mothers of preschoolers and school-aged children. Thus, the comparison with those studies is limited due to the fact that they mostly targeted mothers of infants in Facebook-delivered interventions promoting healthy infant growth or postpartum weight loss and some of those studies conducted outcome assessments remotely [30, 31]. Findings from this study suggest that familiarity with the platform used to deliver health intervention content might be important for retaining participants. Mothers who discontinued their participation were less likely to have read a blog prior to the study and were possibly less inclined to integrate the intervention blog into their routine. Thus, the use of social media platforms that were already part of mothers’ daily social media routines, such as Facebook [22], may be perceived as a more convenient platform to deliver intervention content among this population. Nonetheless, an interesting finding of this study is that fewer participants from the control group discontinued their participation in the trial compared to the intervention group. This may be due to the fact that the delayed access to the blog’s archives containing posts and healthy recipes shared by the RD-blogger may have motivated mothers in the control group to pursue their participation in the study. Indeed, a previous qualitative study [45] demonstrated a high interest of women for the use of blogs for self-management education and social support to help improve diet-related lifestyles and provide valuable interactions with a RD, and findings from this study suggest that this could also apply to mothers of young children.

#### Lesson #5: The effectiveness of retention strategies in social media-delivered dietary interventions should be investigated

The design of this study does not allow for inferring a causal link between the retention strategies that were used and changes in retention rates. Those strategies included employing dedicated research staff who provided flexible appointments at moments that were most convenient for mothers (i.e. early morning, daytime or end of the day), reimbursing parking fees, and allowing mothers to fill web-based questionnaires for outcome assessment at home. Nonetheless, like other clinical researchers [70, 71], those strategies were thought to have been useful assets to reduce the burden associated with in-person meetings and questionnaire completion. One possible way to measure the effectiveness of retention strategies could be to conduct a Study Within a Trial [72, 73] that would allow the evaluation of alternative ways of doing a trial such as recruiting participants and helping them stay in the study.

### Limitations of this study

This study was subject to some limitations that must be acknowledged. First, no data regarding where mothers had heard of the study were obtained for almost 10% of mothers who were screened for eligibility. This could affect the conclusions drawn regarding the reach of each recruitment strategy. Second, results from this study may not be generalized to all mothers of preschoolers and school-aged children, as the sample was composed of well-educated Caucasian women earning high family annual incomes. Therefore, future studies should examine recruitment and retention rates of socioeconomically disadvantaged mothers to serve as a proxy for interest for social media-delivered healthy eating interventions among this population. Last, findings regarding comparison between the characteristics of mothers recruited using Facebook advertisements and those recruited using traditional methods should be interpreted with caution given the unequal sample sizes of the two groups.

## Conclusions

This is the first detailed description of the recruitment and the retention strategies that were used for delivering a healthy eating intervention through a blog in mothers of preschoolers and school-aged children. The planned sample size target was not reached and greater than anticipated losses to follow up were experienced, thus highlighting various challenges associated with interest and acceptability of the intervention format in this population. Recommendations for future research include exploring mothers’ perceptions and preferences regarding social media platforms, including healthy eating blogs and Facebook, to tailor efficient social media recruitment and intervention delivery, considering using social media platforms which are integral part of mothers’ daily social media routine, and providing remote outcome assessments to promote participant retention.

## Supplementary information

**Additional file 1.** Consolidated Standards of Reporting Trials 2010 (CONSORT) checklist.

**Additional file 2.** Comparison of the characteristics at baseline of mothers recruited using Facebook and traditional strategies.

**Additional file 3.** Comparison of the characteristics at baseline of mothers who completed and those who withdrew from the study prior to the end of the 6-month intervention and the 12-month follow-up outcome assessment.

## Data Availability

The datasets used and/or analysed during the current study are available from the corresponding author on reasonable request.

## Abbreviations

RCT: Randomized controlled trial
RD: Registered dietitian
