# Supplementary material for: Recruitment and retention of mothers of preschoolers and school-aged children in a social media-delivered healthy eating intervention: lessons learned from a randomized controlled trial
Source: Trials. 2020 Aug 10;21:706. doi: 10.1186/s13063-020-04628-0 (PMC7418391; doi:10.1186/s13063-020-04628-0)
Supplement: Supplementary file 2 — Additional file 2. Comparison of the characteristics at baseline of mothers recruited using Facebook and traditional strategies. [file 13063_2020_4628_MOESM2_ESM.docx]

**Additional file 2**

Characteristics at baseline of mothers recruited using Facebook (n=3) and traditional strategies (n=78)

|  |  | **Recruitment modality** | | | |  |
| --- | --- | --- | --- | --- | --- | --- |
|  |  | **Facebook (n=3)** | | **Traditional (n=78)** | | ***p value*** |
| **Anthropometric measures** |  | **Mean** | **SD** | **Mean** | **SD** |  |
| Body weight (kg) |  | 84.8 | 17.6 | 70.1 | 13.4 | 0.07 |
| Body mass index (kg/m^2^) |  | 29.6 | 3.9 | 26.2 | 4.9 | 0.14 |
| Waist circumference (cm) |  | 101.7 | 12.0 | 88.1 | 11.2 | 0.06 |
| **Sociodemographic characteristics** |  | **Mean** | **SD** | **Mean** | **SD** |  |
| Age of mother s(years) |  | 34.7 | 8.1 | 37.6 | 6.7 | 0.48 |
| Age of children (years) |  | 3.3 | 1.5 | 8.0 | 3.2 | 0.03 |
|  |  | **n** | **%** | **n** | **%** |  |
| Number of children in mothers' care |  |  |  |  |  | 1.00 |
|  | 1 | 1 | 33 | 21 | 27 |  |
|  | 2 | 1 | 33 | 37 | 47 |  |
|  | 3 and more | 1 | 33 | 20 | 26 |  |
| Ethnicity^a^ |  |  |  |  |  | 1.00 |
|  | Caucasian | 3 | 100 | 70 | 91 |  |
|  | Black | 0 | 0 | 2 | 3 |  |
|  | Latin American | 0 | 0 | 2 | 3 |  |
|  | Arab | 0 | 0 | 3 | 4 |  |
| Marital Status |  |  |  |  |  | 0.33 |
|  | Married or in a common-law relationship | 2 | 67 | 69 | 88 |  |
|  | Separated, divorced, widowed or single | 1 | 33 | 9 | 12 |  |
| Highest level of education completed |  |  |  |  |  |  |
|  | Secondary | 0 | 0 | 4 | 5 |  |
|  | College^b^ | 1 | 33 | 17 | 22 |  |
|  | University^b^ | 2 | 67 | 57 | 73 |  |
| Working Status |  |  |  |  |  | 1.00 |
|  | Full-time job or Full-time student | 3 | 100 | 63 | 81 |  |
|  | Not studying or working full-time | 0 | 0 | 15 | 19 |  |
| Family income (CAN $) |  |  |  |  |  | 0.13 |
|  | 0 - 49,999 | 0 | 0 | 14 | 18 |  |
|  | 50,000 - 99,999 | 3 | 100 | 28 | 36 |  |
|  | 100,000 or more | 0 | 0 | 36 | 46 |  |
| Internet use characteristics |  |  |  |  |  |  |
|  | Read a blog before | 3 | 100 | 63 | 81 | 1.00 |
|  | Read a nutrition blog before^c^ | 2 | 67 | 42 | 67 | 1.00 |
|  | Commented on a blog before^c^ | 2 | 67 | 12 | 19 | 0.11 |
|  | Read comments on a blog before^c^ | 1 | 33 | 51 | 81 | 0.11 |
|  | Use other social media platforms (Facebook, Twitter, etc.) | 3 | 100 | 70 | 90 | 1.00 |
|  | Use the Internet as a source for general health information | 2 | 67 | 47 | 60 | 1.00 |
|  | Use the Internet as a source for nutrition information | 2 | 67 | 58 | 74 | 1.00 |
|  | Use the Internet as a source for recipes | 1 | 33 | 60 | 77 | 0.15 |

SD: Standard deviation.

^a^ n=77 in the Traditional group due to one missing value for ethnicity.

^b^ In Canada, College refers to a post Secondary School degree undertaken prior to University.

^c^ Among those who have read a blog before (n=67).
