# Supplementary material for: Recruitment and retention of mothers of preschoolers and school-aged children in a social media-delivered healthy eating intervention: lessons learned from a randomized controlled trial
Source: Trials. 2020 Aug 10;21:706. doi: 10.1186/s13063-020-04628-0 (PMC7418391; doi:10.1186/s13063-020-04628-0)
Supplement: Supplementary file 3 — Additional file 3. Comparison of the characteristics at baseline of mothers who completed and those who withdrew from the study prior to the end of the 6-month intervention and the 12-month follow-up outcome assessment. [file 13063_2020_4628_MOESM3_ESM.docx]

**Additional file 3**

Characteristics at baseline of mothers who completed (n=62) and those who withdrew from the study prior to the end of the 6-month intervention (n=22)

|  |  | **Completers**  **at 6 months (n=62)** | | **Non-completers at 6 months (n=22)** | | ***p value*** |
| --- | --- | --- | --- | --- | --- | --- |
| **Anthropometric measures** |  | **Mean** | **SD** | **Mean** | **SD** |  |
| Body weight (kg) |  | 69.6 | 13.1 | 72.9 | 15.0 | 0.33 |
| Body mass index (kg/m^2^) |  | 26.0 | 4.8 | 27.2 | 5.2 | 0.29 |
| Waist circumference (cm) |  | 88.3 | 11.8 | 89.5 | 10.3 | 0.66 |
| **Sociodemographic characteristics** |  | **Mean** | **SD** | **Mean** | **SD** |  |
| Age of mothers (years) |  | 38.1 | 6.4 | 36.0 | 7.4 | 0.21 |
| Age of children (years) |  | 7.8 | 3.2 | 7.9 | 3.6 | 0.88 |
|  |  | **n** | **%** | **n** | **%** |  |
| Number of children in mothers' care |  |  |  |  |  | 0.02 |
|  | 1 | 11 | 18 | 12 | 55 |  |
|  | 2 | 33 | 53 | 7 | 32 |  |
|  | 3 and more | 18 | 29 | 3 | 14 |  |
| Ethnicity^a^ |  |  |  |  |  | 0.03 |
|  | Caucasian | 58 | 95 | 18 | 82 |  |
|  | Black | 1 | 2 | 1 | 5 |  |
|  | Latin American | 2 | 3 | 0 | 0 |  |
|  | Arab | 0 | 0 | 3 | 14 |  |
| Marital Status |  |  |  |  |  | 0.47 |
|  | Married or in a common-law relationship | 55 | 89 | 18 | 82 |  |
|  | Separated, divorced, widowed or single | 7 | 11 | 4 | 18 |  |
| Highest level of education completed |  |  |  |  |  | 0.07 |
|  | Secondary | 4 | 6 | 0 | 0 |  |
|  | College^b^ | 11 | 18 | 9 | 41 |  |
|  | University^b^ | 47 | 76 | 13 | 59 |  |
| Working Status |  |  |  |  |  | 0.34 |
|  | Full-time job or Full-time student | 52 | 84 | 16 | 73 |  |
|  | Not studying or working full-time | 10 | 16 | 6 | 27 |  |
| Family income (CAN $) |  |  |  |  |  | 0.02 |
|  | 0 - 49,999 | 8 | 13 | 9 | 41 |  |
|  | 50,000 - 99,999 | 24 | 39 | 7 | 32 |  |
|  | 100,000 or more | 30 | 48 | 6 | 27 |  |
| Internet use characteristics |  |  |  |  |  |  |
|  | Read a blog before | 54 | 87 | 13 | 59 | 0.01 |
|  | Read a nutrition blog before^c^ | 35 | 65 | 10 | 77 | 0.52 |
|  | Commented on a blog before^c^ | 13 | 24 | 1 | 8 | 0.27 |
|  | Read comments on a blog before^c^ | 44 | 81 | 9 | 69 | 0.45 |
|  | Use other social media platforms (Facebook, Twitter, etc.) | 55 | 89 | 20 | 91 | 1.00 |
|  | Use the Internet as a source for general health information | 35 | 56 | 15 | 68 | 0.34 |
|  | Use the Internet as a source for nutrition information | 44 | 72 | 19 | 86 | 0.18 |
|  | Use the Internet as a source for recipes | 49 | 79 | 15 | 68 | 0.30 |

SD: Standard deviation.

^a^ n=61 in the Completers group due to one missing value for ethnicity.

^b^ In Canada, College refers to a post Secondary School degree undertaken prior to University.

^c^ Among mothers who have read a blog before (n=67).

Characteristics at baseline of mothers who completed (n=56) and those who withdrew from the study prior to the 12-month follow-up outcome assessment (n=28)

|  |  | **Completers**  **at 12 months**  **(n=56)** | | **Non-completers at 12 months (n=28)** | | ***p* value** |
| --- | --- | --- | --- | --- | --- | --- |
| **Anthropometric measures** |  | **Mean** | **SD** | **Mean** | **SD** |  |
| Body weight (kg) |  | 70.3 | 13.5 | 70.7 | 14.0 | 0.90 |
| Body mass index (kg/m^2^) |  | 26.3 | 4.9 | 26.3 | 5.0 | 0.98 |
| Waist circumference (cm) |  | 88.9 | 12.0 | 88.1 | 10.2 | 0.79 |
| **Sociodemographic characteristics** |  | **Mean** | **SD** | **Mean** | **SD** |  |
| Age of mothers (years) |  | 38.6 | 5.7 | 35.5 | 8.0 | 0.08 |
| Age of children (years) |  | 8.0 | 3.1 | 7.4 | 3.6 | 0.58 |
|  |  | **n** | **%** | **n** | **%** |  |
| Number of children in mothers' care |  |  |  |  |  | 0.001 |
|  | 1 | 8 | 14 | 15 | 54 |  |
|  | 2 | 31 | 55 | 9 | 32 |  |
|  | 3 and more | 17 | 30 | 4 | 14 |  |
| Ethnicity^a^ |  |  |  |  |  | 0.06 |
|  | Caucasian | 52 | 95 | 24 | 86 |  |
|  | Black | 1 | 2 | 1 | 4 |  |
|  | Latin American | 2 | 4 | 0 | 0 |  |
|  | Arab | 0 | 0 | 3 | 11 |  |
| Marital Status |  |  |  |  |  | 0.49 |
|  | Married or in a common-law relationship | 50 | 89 | 23 | 82 |  |
|  | Separated, divorced, widowed or single | 6 | 11 | 5 | 18 |  |
| Highest level of education completed |  |  |  |  |  | 0.11 |
|  | Secondary | 4 | 7 | 0 | 0 |  |
|  | College^b^ | 10 | 18 | 10 | 36 |  |
|  | University^b^ | 42 | 75 | 18 | 64 |  |
| Working Status |  |  |  |  |  | 0.33 |
|  | Full-time job or Full-time student | 47 | 84 | 21 | 75 |  |
|  | Not studying or working full-time | 9 | 16 | 7 | 25 |  |
| Family income (CAN $) |  |  |  |  |  | 0.01 |
|  | 0 - 49,999 | 6 | 11 | 11 | 39 |  |
|  | 50,000 - 99,999 | 22 | 39 | 9 | 32 |  |
|  | 100,000 or more | 28 | 50 | 8 | 29 |  |
| Internet use characteristics |  |  |  |  |  |  |
|  | Read a blog before | 48 | 86 | 19 | 68 | 0.05 |
|  | Read a nutrition blog before^c^ | 30 | 63 | 15 | 79 | 0.20 |
|  | Commented on a blog before^c^ | 12 | 25 | 2 | 11 | 0.19 |
|  | Read comments on a blog before^c^ | 38 | 79 | 15 | 79 | 1.00 |
|  | Use other social media platforms (Facebook, Twitter, etc.) | 50 | 89 | 25 | 89 | 1.00 |
|  | Use the Internet as a source for general health information | 31 | 55 | 19 | 68 | 0.27 |
|  | Use the Internet as a source for nutrition information | 40 | 73 | 23 | 82 | 0.34 |
|  | Use the Internet as a source for recipes | 45 | 80 | 19 | 68 | 0.20 |

SD: Standard deviation.

^a^ n=55 in the Completers group due to one missing value for ethnicity.

^b^ In Canada, College refers to a post Secondary School degree undertaken prior to University.

^c^ Among mothers who have read a blog before (n=67).
